# Intragastric Balloon-Induced Pancreatitis in a 21-Year-Old Male: A Case-Based Review

**DOI:** 10.7759/cureus.102273

**Published:** 2026-01-25

**Authors:** Yousif Alabboudi, Mallika Mehrotra

**Affiliations:** 1 General Surgery, Rashid Hospital, Dubai, ARE; 2 Medicine, Mohammed Bin Rashid University of Medicine and Health Sciences, Dubai, ARE

**Keywords:** gastric complications, igb complication, igb-induced pancreatitis, intragastric balloon (igb), recurrent acute pancreatitis

## Abstract

Intragastric balloon (IGB) placement is a temporary, minimally invasive weight loss intervention that is generally well tolerated; however, it may be associated with complications. While most adverse effects are mild, acute pancreatitis is a rare but potentially serious complication, most commonly attributed to extrinsic pancreatic compression.

We report a case of acute pancreatitis developing eight weeks after IGB insertion in a 21-year-old male. The patient presented with severe epigastric pain radiating to the back and repeated vomiting following binge eating. Laboratory testing revealed markedly elevated serum lipase at 3,217 U/L (reference <60 U/L) and elevated CRP at 10.7 mg/L, with normal liver function tests, calcium, and triglyceride levels. Contrast-enhanced computed tomography demonstrated diffuse pancreatic enlargement with peripancreatic fat stranding, consistent with acute interstitial edematous pancreatitis. The Bedside Index of Severity in Acute Pancreatitis score was 0. With gallstones, alcohol use, and metabolic causes excluded, pancreatitis was attributed to extrinsic pancreatic compression from the IGB, precipitated by gastric distention. The patient improved with conservative management and was discharged after one week.

Acute pancreatitis is a rare but serious complication of IGB placement. This case emphasizes the need for clinicians to maintain a high index of suspicion in patients presenting with epigastric pain after the procedure, particularly following dietary indiscretion.

## Introduction

Obesity (BMI >30) is a growing global health concern, often associated with comorbidities such as type 2 diabetes, hypertension, and cardiovascular disease [1]. While various surgical and non-surgical interventions exist, minimally invasive procedures are increasingly favored for weight management.

The intragastric balloon (IGB) is an endoscopic weight-loss device typically used when conservative measures fail or as a bridge to bariatric surgery [2]. Although less invasive than bariatric surgery, IGB therapy carries potential complications. Common adverse effects include transient nausea and vomiting, whereas serious complications may consist of gastric ulceration, balloon migration or rupture, perforation, and acute pancreatitis. Since 2016, multiple cases of IGB-associated pancreatitis have been reported to the U.S. Food and Drug Administration, with incidence varying by balloon type [3,4].

Several mechanisms have been proposed for IGB-induced pancreatitis. The most common involves direct mechanical compression of the pancreas by the distended stomach, which may impair perfusion and trigger inflammation. Extrinsic compression of the duodenum or pancreatic duct can obstruct pancreatic outflow. At the same time, additional contributing factors include local ischemia, increased intragastric pressure after dietary indiscretion, and mechanical irritation from balloon overfilling or migration [5-10]. These factors often act together, explaining why most cases occur within weeks of insertion.

We report a case of acute pancreatitis following IGB placement in a young adult, highlighting a rare but serious complication and emphasizing the importance of early recognition. This case contributes to the literature by illustrating the temporal relationship between dietary indiscretion, gastric distention, and pancreatic injury, reinforcing the need for vigilance in patients presenting with epigastric pain.

## Case presentation

A 21-year-old male presented to the emergency department with a five-hour history of severe epigastric pain radiating to the back, accompanied by repeated vomiting of bile-free gastric contents. He denied systemic, cardiopulmonary, bowel, or urinary symptoms. This episode followed binge eating on fast food.

He had two prior self-limited episodes of epigastric pain following binge eating at two and four weeks post-insertion, which resolved without medical evaluation. His medical history was notable for class II obesity (BMI 35.8 kg/m²) [7,8]. He reported no alcohol consumption or use of medications.

On examination, he was afebrile and hemodynamically stable. He was alert and oriented but mildly distressed. The abdomen was non-distended with no cutaneous changes. Palpation revealed localized epigastric tenderness with a negative Murphy’s sign. Bowel sounds were present and normal. The patient’s laboratory results at admission are summarized in Table 1.

**Table 1:** Laboratory results of this patient at admission CRP: C-reactive protein, ALT: alanine aminotransferase, AST: aspartate aminotransferase

Test	Result	Reference range
White blood cell count	11.0 ×10⁹/L	4-11 ×10⁹/L
Hemoglobin	15.4 g/dL	12-16 g/dL
Hematocrit	45.6%	36-46%
Platelets	310 ×10⁹/L	150-400 ×10⁹/L
Creatinine	0.98 mg/dL	0.6-1.2 mg/dL
CRP	10.7 mg/L	<5 mg/L
ALT	20 U/L	<40 U/L
AST	17 U/L	<40 U/L
Alkaline phosphatase	58 U/L	30-120 U/L
Total bilirubin	0.18 mg/dL	<1.2 mg/dL
Serum lipase	3,217 U/L	<60 U/L

Laboratory testing revealed a markedly elevated serum lipase of 3,217 U/L (reference <60 U/L), confirming acute pancreatic injury, and an elevated CRP of 10.7 mg/L, indicating systemic inflammation. Other labs, including liver function tests, serum calcium, and triglycerides, were within normal limits (Table 1).

**Figure 1:**
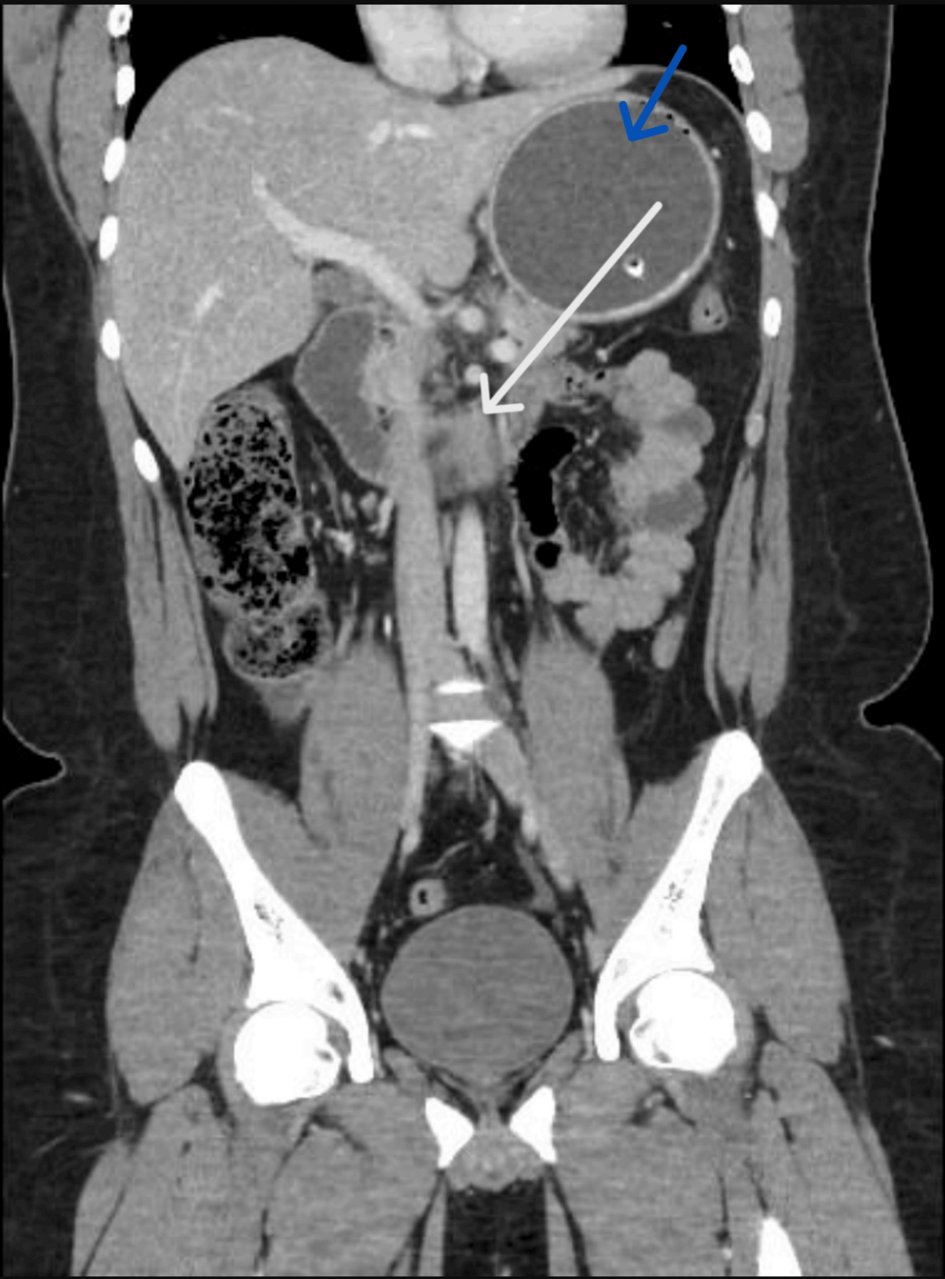
Contrast-enhanced CT of the abdomen showing diffuse enlargement of the pancreas with surrounding peripancreatic fat stranding, consistent with acute interstitial edematous pancreatitis (white arrow). The IGB is visualized in situ (blue arrow) CT: computed tomography, IGB: intragastric balloon

A plain abdominal X-ray had confirmed the balloon in situ, without evidence of bowel obstruction or pneumoperitoneum. The contrast-enhanced abdominal CT scan demonstrated diffuse pancreatic enlargement with surrounding peripancreatic fat stranding, consistent with acute interstitial edematous pancreatitis (Figure 1).

A diagnosis of acute pancreatitis was made, with a Bedside Index of Severity in Acute Pancreatitis (BISAP) score of 0 [9]. Given that gallstones, alcohol, and hypertriglyceridemia were excluded, the likely etiology was extrinsic pancreatic compression by the IGB, precipitated by gastric distention after a heavy meal.

The patient was admitted under general surgery and managed conservatively with intravenous fluids and analgesia. As the episodes were clearly triggered by dietary indiscretion, the patient elected to continue with the balloon in place until his intended weight loss goal was achieved, with dietary counselling to prevent recurrence. He was discharged after one week of clinical improvement, with follow-up arranged to monitor for pancreatitis-related complications.

## Discussion

IGB therapy has been widely used for weight management over the past four decades, with refinements in balloon design and endoscopic techniques improving safety and tolerability [1,2,7-11]. Despite this, IGB placement carries potential complications ranging from transient nausea and vomiting to serious outcomes such as balloon migration, gastric perforation, and acute pancreatitis [2,8].

Acute pancreatitis is a rare but increasingly recognized complication of IGB therapy. Since the first report in 2008, multiple case reports and FDA post-marketing alerts have documented over 50 occurrences [3,5-12]. The Manufacturer and User Facility Device Experience (MAUDE) database review further identified pancreatitis as one of the most frequent severe adverse events associated with IGB therapy, highlighting the need for prompt recognition and reporting [4]. Presentations vary in severity and timing. Some cases were managed conservatively after balloon removal [9], while others highlighted mechanical compression and balloon overinflation as primary triggers [5,6]. Additional reports describe pancreatic tail compression and recurrent episodes due to balloon migration [11,13], underscoring the need for vigilance in patients presenting with abdominal pain.

Pathophysiology of IGB-induced pancreatitis involves multiple, often interacting mechanisms. Direct pancreatic compression by a distended stomach can impair perfusion and trigger local inflammation. Extrinsic compression of the duodenum or pancreatic duct may obstruct pancreatic outflow, leading to enzyme accumulation and autodigestion. Balloon overinflation or migration can exacerbate mechanical pressure, while gastric distention after dietary indiscretion increases intraluminal pressure, precipitating recurrent pancreatic injury [2,7,11,12]. These mechanisms act synergistically, explaining the variable onset and severity observed in reported cases.

Our case is distinctive for recurrent pancreatitis in a young, otherwise healthy male, consistently triggered by dietary indiscretion. Unlike most reported cases where the balloon was removed after a single episode [2,7,9,11], our patient opted to continue therapy with strict dietary modification and close follow-up. While this carries potential risks of recurrent pancreatitis, local inflammation, or ductal injury, it may be considered in carefully selected patients with a clear understanding of the complications.

From a clinical standpoint, any patient with an IGB presenting with abdominal pain should be evaluated promptly for pancreatitis [14]. Serum lipase and CRP are key diagnostic markers, and the BISAP score is a validated tool for assessing severity and guiding management [9,15]. In our patient, a BISAP score of 0 correlated with a mild course, supporting conservative management.

In summary, IGB therapy remains an effective weight-loss modality, but acute pancreatitis is a rare, potentially recurrent complication. Clinicians should maintain a high index of suspicion, counsel patients on dietary habits to minimize risk, and individualize follow-up or balloon removal based on recurrence and severity. This case provides a clinical pearl: recurrent IGB-induced pancreatitis may be considered for conservative management under careful monitoring in highly selected patients after shared decision-making, highlighting the importance of patient-centered decision-making. Future studies and pooled analyses, including MAUDE data, are needed to clarify risk factors, preventive strategies, and optimal management protocols.

## Conclusions

This case highlights IGB-induced acute pancreatitis as a rare but clinically significant complication, even in young and otherwise healthy individuals. The recurrence of symptoms in temporal association with binge eating underscores the role of gastric overdistension in precipitating pancreatic compression. In carefully selected patients, conservative management with dietary modification and close follow-up may be a feasible approach, although vigilance for recurrence is essential. Clinicians should maintain a high index of suspicion for pancreatitis in patients with IGB presenting with abdominal pain, and patient education on meal habits and portion control is critical to minimize risk.

Future research, including larger studies and pooled analyses, is needed to clarify risk factors, preventive strategies, and optimal management pathways for IGB-induced pancreatitis.
